# HIV Infection as Risk Factor for Death among Hospitalized Persons with Candidemia, South Africa, 2012–2017

**DOI:** 10.3201/eid2706.210128

**Published:** 2021-06

**Authors:** Nelesh P. Govender, Jim Todd, Jeremy Nel, Mervyn Mer, Alan Karstaedt, Cheryl Cohen, for GERMS-SA

**Affiliations:** University of Cape Town, Cape Town, South Africa (N.P. Govender);; University of the Witwatersrand, Johannesburg (N.P. Govender, J. Nel, M. Mer, A. Karstaedt, C. Cohen);; National Institute for Communicable Diseases, a Division of the National Health Laboratory Service, Johannesburg, South Africa (N.P. Govender, C. Cohen);; London School of Hygiene and Tropical Medicine, London, UK (J. Todd)

**Keywords:** *Candida*, candidemia, mortality, sepsis, *Candida albicans*, fungi, HIV/AIDS and other retroviruses, South Africa

## Abstract

We determined the effect of HIV infection on deaths among persons >18 months of age with culture-confirmed candidemia at 29 sentinel hospitals in South Africa during 2012–2017. Of 1,040 case-patients with documented HIV status and in-hospital survival data, 426 (41%) were HIV-seropositive. The in-hospital case-fatality rate was 54% (228/426) for HIV-seropositive participants and 37% (230/614) for HIV-seronegative participants (crude odds ratio [OR] 1.92, 95% CI 1.50–2.47; p<0.001). After adjusting for relevant confounders (n = 907), mortality rates were 1.89 (95% CI 1.38–2.60) times higher among HIV-seropositive participants than HIV-seronegative participants (p<0.001). Compared with HIV-seronegative persons, the stratum-specific adjusted mortality OR was higher among HIV-seropositive persons not managed in intensive care units (OR 2.27, 95% CI 1.47–3.52; p<0.001) than among persons who were (OR 1.56, 95% CI 1.00–2.43; p = 0.05). Outcomes among HIV-seropositive persons with candidemia might be improved with intensive care.

## Abstract 2

HIV-seropositive persons demonstrated increased adjusted risk for 30-day mortality and should be evaluated for intensive care.

*Candida* is a common cause of healthcare-associated bloodstream infections in South Africa; the estimated national incidence risk in 2016–2017 was 84 (95% CI 81–86) cases per 100,000 hospital admissions (*1*). This rate is ≈10-fold higher than that reported in the United States (*2*). The death rate among patients with *Candida* bloodstream infections is high and associated with such factors as confirmed or presumed gastrointestinal source of infection, lack of source control, shorter time to positivity of blood cultures, inappropriate or delayed empiric antifungal treatment, lack of consultation with an infectious disease physician, severe sepsis or septic shock, and severity of underlying conditions (*3*–*5*). The crude mortality rate associated with candidemia was 43% in South Africa in 2016–2017 (*1*). In 2 small case series of HIV-seropositive patients with candidemia (Spain, n = 37; Italy, n = 38), the crude mortality rate was ≈60% (*6*,*7*). HIV infection may be a risk factor for death among persons with culture-confirmed candidemia. In a 2014 population-based surveillance study in Spain, 16 (2%) of 752 patients with candidemia were HIV-seropositive (*8*). Although HIV infection was not associated with death on univariable analysis (OR 0.51, 95% CI 0.07–3.93), the study was underpowered. In contrast, in a United States cohort study of 446 adults with candidemia, 22 (5%) were HIV-seropositive, and HIV infection was associated with a 2-fold increased adjusted hazard of 30-day mortality (hazard ratio 2.02, 95% CI 1.11–3.72) (*9*). This effect of HIV infection on death rates may be mediated by factors related to the host (e.g., neutropenia or neutrophil dysfunction in persons with advanced HIV disease) or the fungal pathogen (infection caused by *Candida* species other than *C. albicans* or by >1 *Candida* spp.) (*6*,*7*,*10*–*12*). Such an association has not been described in a high HIV prevalence setting. By using data from a 6-year sentinel surveillance study at hospitals in South Africa, we examined the effect of HIV infection on the risk for 30-day mortality among persons with candidemia.

## Materials and Methods

### Study Population

We included persons >18 months of age with an episode of culture-confirmed candidemia identified at 29 sentinel hospitals in South Africa during January 1, 2012–December 31, 2017. The HIV prevalence among inpatients is high at these large urban tertiary-academic or regional acute-care hospitals (e.g., 60% at a Cape Town district hospital in 2012–2013) (*13*). A case was defined as illness in a person in whom *Candida* spp. was cultured from blood at a laboratory providing diagnostic pathology services to a sentinel hospital. An episode was defined as a 30-day period from the date of the first positive *Candida* culture. Any positive blood cultures after this period defined a recurrent episode and thus a new case. We excluded children <18 months of age because we did not have HIV PCR results to confirm an HIV diagnosis for a sufficient number of persons.

### Surveillance Methods

Cases were reported from public-sector and private-sector pathology laboratories. In general, blood cultures were collected if patients had clinical features of sepsis, including tachycardia, tachypnea, increased or subnormal temperature, a change in sensorium, hypotension, or prostration (*14*). Viable *Candida* isolates were submitted to a reference laboratory for species-level identification and antifungal susceptibility testing, as previously described (*1*). Trained nurses or pharmacists identified culture-confirmed cases in the laboratory and interviewed prospectively enrolled participants or their next of kin or reviewed medical charts or electronic laboratory records. Data were collected through a standardized case report form. The main explanatory variable was the participant’s HIV infection status, which was determined during the admission for candidemia. HIV status was self-reported during participant interviews or abstracted from the inpatient chart, a child’s outpatient immunization card, or laboratory records. An in-hospital outcome with a date of outcome was determined at the end of admission to the acute-care hospital or if the participant was transferred to a step-down facility; this outcome was determined at the end of that admission. Outcome was recorded as alive at 30 days for prolonged admissions of >30 days after first positive blood culture. Underlying or immediate cause of death was not recorded. Surveillance officers recorded whether a person had been admitted to the intensive care unit (ICU) at any time during hospitalization, but the specific reason for and dates of ICU admission or discharge were not captured. We calculated a quick Pitt score, an abbreviated version of the Pitt bacteremia score, as the sum of individual scores for body temperature of <35°C (1 point), systolic blood pressure of <90 mm Hg (1 point), cardiac arrest (1 point), mechanical ventilation (1 point ), and altered mental status (1 point) on the day of candidemia diagnosis (*15*,*16*).

### Data Analysis

We used participant identifiers to remove duplicate records. We described the characteristics of the study participants by using descriptive statistics. To determine risk factors for death, we used classical Mantel-Haenszel methods to calculate crude case fatality ratio, odds ratio (OR), and 95% CI for HIV status and each of the potential confounders (Appendix). We did not assume that individual participant-level outcomes were statistically independent in this sentinel surveillance study. We therefore used a random-effects logistic regression analysis to explicitly model between-cluster variation at sentinel sites and simultaneously adjust for participant-level confounders (*17*) (Appendix). We treated the follow-up period as a fixed 30-day period. We also explored the dose-response effect of HIV on death rates. For this analysis, participants’ HIV infection status was recoded as an ordinal variable: HIV-seronegative, HIV-seropositive without advanced immunosuppression (CD4 count >200 cells/µL), and HIV-seropositive with advanced immunosuppression (CD4 count <200 cells/µL). Before conducting the analysis, we hypothesized that the effect of HIV infection on death rates would differ among participants with candidemia managed in an ICU and those who were not admitted to an ICU; therefore, we considered ICU admission as an effect modifier a priori. We included participants with recorded dates of positive *Candida* specimen collection and 30-day outcome in a Kaplan-Meier survival analysis. For 67 participants with an outcome date that coincided exactly with the specimen collection date, we recoded their time-to-outcome to 0.5 days. We explored the association between HIV status and ICU admission by classical and multivariable random effects logistic regression analyses as a post-hoc analysis to explore reasons for the main results.

### Ethics

For GERMS-SA surveillance, annual ethics approvals were sought and obtained from several university ethics committees in South Africa. We also obtained approval from the London School of Hygiene and Tropical Medicine Research Ethics Committee.

## Results

**Figure 1:**
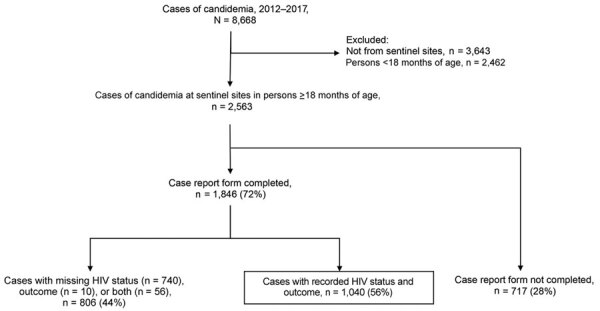
Flowchart demonstrating selection of 1,040 cases of candidemia from a 6-year surveillance period for secondary data analysis, South Africa, 2012–2017.

After deduplication, 8,668 cases were detected by GERMS-SA surveillance during the 6-year period. We excluded 3,643 cases diagnosed at non–sentinel sites and 2,462 cases among infants and children <18 months of age. Of 2,563 cases diagnosed at sentinel sites, a case report form had been completed for 1,846 (72%) (Figure 1). Of those, we retained 1,040 cases with both HIV status and outcome data (56%). We noted differences in age, sex, year of diagnosis, province in which the diagnosis was made, and *Candida* species among 717 sentinel-site cases with missing case report forms, 806 cases with completed case report forms but missing HIV status or outcome data, and the 1,040 cases included in the final analysis (Appendix Table 1).

**Table 1:** Comparison of baseline characteristics by HIV infection status among 1,040 persons with culture-confirmed candidemia at sentinel hospitals, South Africa, 2012–2017*

Variable	No. patients†	HIV-seronegative, n = 614	HIV-seropositive, n = 426	p value‡
Median age, y (IQR)	1,037	38.8 (16.1–57.6)	36.4 (27.6–46.1)	0.05§
Age group, y	1,037			
<18	226	161 (71)	65 (29)	<0.001
18–44	427	190 (45)	237 (55)	
45–64	283	173 (61)	110 (39)	
>65	101	88 (87)	13 (13)	
Missing	3			
Sex	1,040			
F	498	272 (55)	226 (45)	0.005
M	542	342 (63)	200 (37)	
Province	1,040			
Other province	495	297 (60)	198 (40)	
Gauteng	545	317 (58)	228 (42)	0.55
Year	1,040			
2012	139	96 (69)	43 (31)	<0.001
2013	185	117 (63)	68 (37)	
2014	100	48 (48)	52 (52)	
2015	91	45 (49)	46 (51)	
2016	273	175 (64)	98 (36)	
2017	252	133 (53)	119 (47)	
Community-onset infection, <72 h preadmission	1,035			
No	747	454 (61)	293 (39)	0.05
Yes	288	156 (54)	132 (46)	
Missing	5			
ICU admission	1,023			
No	509	262 (51)	247 (49)	<0.001
Yes	514	339 (66)	175 (34)	
Missing	17			
CVC in situ	1,011			
No	466	243 (52)	223 (48)	<0.001
Yes	545	354 (65)	191 (35)	
Missing	29			
Total parenteral nutrition	1,004			
No	759	425 (56)	334 (44)	<0.001
Yes	245	167 (68)	78 (32)	
Missing	36			
Quick Pitt score >2¶	652			
No	503	278 (55)	225 (45)	0.27
Yes	149	90 (60)	59 (40)	
Missing	388			
*Candida* species	946			
* C. albicans*	425	221 (52)	204 (48)	<0.001
Other *Candida* species#	521	336 (65)	185 (35)	
Missing	94			
Mixed *Candida* species	1,040			
No	1,007	590 (59)	417 (41)	0.11**
Yes	33	24 (73)	9 (27)	
*Candida* species resistant to fluconazole, voriconazole, or an echinocandin††	764			
No	610	345 (57)	265 (43)	<0.001
Yes	154	114 (74)	40 (26)	
Missing	276			
Receipt of systemic antifungal treatment	1,010			
No	310	153 (49)	157 (51)	<0.001
Yes	700	442 (63)	258 (37)	
Missing	30			
Inappropriate antifungal treatment for candidemia‡‡	419			
No	381	247 (65)	134 (35)	0.21**
Yes	38	29 (76)	9 (24)	
Missing	621			
Removal of CVC	488			
No	127	75 (59)	52 (41)	0.08
Yes	361	244 (68)	117 (32)	
Missing	552			

*Values are no. (%) unless otherwise indicated. CVC, central venous catheter; ICU, intensive care unit. †Total numbers for each category indicate number of patients for whom that information was available. ‡p values by Pearson’s χ^2^ test unless otherwise indicated.  §By Wilcoxon rank-sum test.  ¶Quick Pitt score was calculated as the sum of individual scores for temperature <35^o^C (1), systolic blood pressure <90 mm Hg (1), cardiac arrest (1), mechanical ventilation (1), and altered mental status (1) at time of candidemia diagnosis.  #Other *Candida* species in HIV-seronegative participants (*Candida parapsilosis*, n = 112; *Candida glabrata*, n = 84; *Candida auris*, n = 27; *Candida tropicalis*, n = 22; *Candida krusei*, n = 20; other species, n = 71) and HIV-seropositive participants (*C. parapsilosis*, n = 51; *C. glabrata*, n = 49; *C. tropicalis*, n = 20; *C. auris*, n = 8; *C. krusei*, n = 8; other species, n = 49).  **By Fisher exact test.  ††Resistance was defined based on Clinical and Laboratory Standards Institute *Candida* species–specific breakpoints (*18*).  ‡‡Inappropriate treatment was defined as treatment with an antifungal agent to which the *Candida* species was resistant.

### Description of 1,040 Participants Included in the Analysis

Of 1,040 participants, 542 (52%) were men and boys (Table 1). The median age of 1,037 participants with recorded age was 37 years (interquartile range [IQR] 23–52 years). Of 1,035 participants with available date of specimen collection, 288 (28%) had a positive *Candida* blood culture within 72 hours of hospital admission. Overall, 50% (514/1,023) participants were managed in the ICU during their hospitalization (data on ICU admission were missing for 17 participants). At the time of diagnosis, most (545/1,011, 54%) had a central venous catheter (CVC) in situ, and 24% (245/1,004) were receiving total parenteral nutrition. Of 1,001 participants for whom data was available, 163 (16%) received previous antifungal treatment. A quick Pitt score was calculated for 652/1,040 participants: 319 (49%) had a 0 score, 184 (28%) had a 1 score, 126 (19%) had a 2 score, 20 (3%) had a 3 score, and 3 (1%) had a 4 score (Appendix). Thus, 149/652 (23%) had a quick Pitt score of >2. Of 946 case-patients in which *Candida* species identification was performed at a diagnostic or reference laboratory, 521 (55%) participants were infected with a *Candida* species other than *C. albicans* and the remainder with *C. albicans*; 33 (3%) case-patients had a mixed *Candida* infection. Of 1,010 cases, 700 (69%) received systemic antifungal treatment to treat candidemia (participants might have received >1 antifungal agent: fluconazole [n = 503], amphotericin B [n = 265], echinocandin [n = 91], or voriconazole [n = 17]). Of 764 cases with a known *Candida* species, available antifungal susceptibility data, and applicable Clinical and Laboratory Standards Institute breakpoints (*18*), 154 (20%) were infected with an antifungal-resistant species. Of these 764, antifungal treatment was recorded for 419, and 9% (38/419) had received inappropriate antifungal treatment. Of 488 cases with recorded information, 361 (74%) had their CVC removed after the candidemia diagnosis. In 4% of patients (45/1,040), evidence of complications of candidemia (deep organ involvement) was indicated in medical charts.

### HIV Status and Outcome

Of the 1,040 case-patients, 426 (41%) were HIV-seropositive; we noted several differences in those patients compared with those who were HIV-seronegative (Table 1; Appendix). Among 404 HIV-seropositive persons with available data, 301 (75%) were antiretroviral treatment–experienced. Among 267 participants in whom CD4 count was recorded near the date of the candidemia diagnosis, the median CD4 count was 133 (IQR 42–309) cells/µL; 166/267 (63%) had a CD4 count <200 cells/µL. An additional 35 without a CD4 count had a recorded World Health Organization (WHO) clinical stage of HIV disease. Of these 35 case-patients, illness was WHO stage 3 or 4 in 33 case-patients. Of 141 whose records indicated a recently recorded viral load, 65 (46%) had a viral load of <400 RNA copies/mL. Of the 426 HIV-seropositive persons, 153 (36%) had clinical evidence of HIV-associated wasting. The overall case fatality ratio was 458/1,040 (44%). The case-fatality ratio among HIV-seronegative cases was 37% (230/614) versus 54% (228/426) for HIV-seropositive cases (p<0.001).

### Risk Factors for 30-Day Mortality

The crude 30-day case fatality ratio among HIV-seropositive participants was 1.92 (95% CI 1.50–2.47) times higher than among HIV-seronegative participants (p<0.001) (Table 2). After adjusting for sentinel hospital, age, sex, year of diagnosis, ICU admission, receipt of systemic antifungal treatment, and *Candida* species, the odds of 30-day mortality were still 1.89 (95% CI 1.38–2.60) times higher among HIV-seropositive participants than among HIV-seronegative participants (Table 3); evidence was strong against the null hypothesis (p<0.001). We noted relatively little confounding of the association between HIV status and 30-day mortality by any available explanatory variable. In the final model, differences between hospitals accounted for 3% of the variability in deaths (intracluster correlation coefficient = 0.03; p = 0.003). We found evidence of interaction of HIV status and ICU admission. The stratum-specific mortality OR was larger in the group not managed in an ICU (OR 2.27, 95% CI 1.47–3.52; p<0.001) than those who were admitted to the ICU (OR 1.56, 95% CI 1.00–2.43; p = 0.05), although the 95% CIs overlapped (Table 4). In a dose-response analysis, the adjusted odds of 30-day mortality was 1.90 times higher among HIV-seropositive persons with a CD4 count >200 cells/µL (95% CI 1.13–3.20; p = 0.02) and 2.18 times higher among persons with a CD4 count <200 cells/µL (95% CI 1.39–3.42; p = 0.001) compared with 30-day mortality among HIV-seronegative persons (Appendix Table 4).

**Table 2:** Risk factors for in-hospital death among 1,040 persons with culture-confirmed candidemia at sentinel hospitals, South Africa, 2012–2017*

Variable	No. patients†	No. deaths	Case-fatality ratio, %	Crude OR (95% CI)	LRT p value
HIV status	1,040				
Seronegative	614	230	37	Referent	<0.001
Seropositive	426	228	54	1.92 (1.50–2.47)	
Age group, y	1,037				
<18	226	46	20	Referent	<0.001
18–44	427	199	47	3.42 (2.35–4.97)	
45–64	283	152	54	4.54 (3.04–6.77)	
>65	101	61	60	5.97 (3.57–9.97)	
Missing	3				
Sex	1,040				
F	498	215	43	Referent	0.59
M	542	243	45	1.07 (0.84–1.37)	
Province	1,040				
Other province	495	232	50	Referent	0.21
Gauteng	545	226	46	0.86 (0.67–1.09)	
Year	1,040				
2012	139	52	37	Referent	0.05
2013	185	74	40	1.11 (0.71–1.75)	
2014	100	51	51	1.74 (1.03–2.93)	
2015	91	38	42	1.20 (0.70–2.06)	
2016	273	115	42	1.22 (0.80–1.85)	
2017	252	128	51	1.73 (1.13–2.64)	
Community-onset infection, <72 h preadmission	1,035				
No	747	334	45	Referent	0.38
Yes	288	120	42	0.88 (0.67–1.16)	
Missing	3				
ICU admission	1,023				
No	509	202	40	Referent	0.005
Yes	514	249	48	1.43 (1.11–1.83)	
Missing	17				
CVC in situ	1,011				
No	466	193	41	Referent	0.21
Yes	545	247	45	1.17 (0.91–1.51)	
Missing	29				
Total parenteral nutrition	1,004				
No	759	333	44	Referent	0.70
Yes	245	104	42	0.94 (0.71–1.26)	
Missing	36				
Quick Pitt score >2‡	652				
No	503	188	37	Referent	<0.001
Yes	149	89	60	2.49 (1.71–3.61)	
Missing	388				
*Candida* species	946				
* C. albicans*	425	221	52	Referent	<0.001
Other *Candida* species	521	204	39	0.59 (0.46–0.77)	
Missing	94				
Mixed *Candida* species	1,040				
No	1,007	445	44	Referent	0.58
Yes	33	13	40	0.82 (0.40–1.67)	
*Candida* species resistant to fluconazole, voriconazole, or an echinocandin§	764				
No	610	283	46	Referent	0.35
Yes	154	65	42	0.84 (0.59–1.21)	
Missing	276				
Receipt of systemic antifungal treatment	1,010				
No	310	187	60	Referent	<0.001
Yes	700	254	36	0.37 (0.28–0.49)	
Missing	30				
Inappropriate antifungal treatment for candidemia¶	419				
No	381	140	37	Referent	0.52
Yes	38	16	42	1.25 (0.64–2.46)	
Missing	621				
Removal of CVC	488				
No	127	71	56	Referent	0.001
Yes	361	141	39	0.51 (0.34–0.76)	
Missing	552				

*CVC, central venous catheter; ICU, intensive care unit; LRT, likelihood ratio test; OR, odds ratio.  †Total numbers for each category indicate number of patients for whom that information was available. ‡Quick Pitt score was calculated as the sum of individual scores for temperature <35°C (1), systolic blood pressure <90 mm Hg (1), cardiac arrest (1), mechanical ventilation (1), and altered mental status (1) at time of diagnosis of candidemia.  §Resistance was defined on the basis of Clinical and Laboratory Standards Institute *Candida* species–specific breakpoints (*18*).  ¶Inappropriate treatment was defined as treatment with an antifungal agent to which the *Candida* species was resistant.

**Table 3:** Random-effects multivariable logistic regression analysis of the effect of HIV on in-hospital death by sentinel site, simultaneously adjusted for potential confounders, among 907 persons with candidemia, South Africa, 2012–2017*

Variable	Summary aOR for death (95% CI)	Wald p value
HIV status		
Seronegative	Referent	
Seropositive	1.89 (1.38–2.60)	<0.001
Age group, y		
<18	Referent	
18–44	2.55 (1.66–3.93)	<0.001
45–64	3.48 (2.21–5.49)	<0.001
>65	6.47 (3.61–11.61)	<0.001
Sex		
F	Referent	
M	1.27 (0.95–1.70)	0.11
Year		
2012	Referent	
2013	1.26 (0.72–2.19)	0.42
2014	1.34 (0.67–2.68)	0.40
2015	1.17 (0.58–2.33)	0.66
2016	1.08 (0.63–1.86)	0.77
2017	1.53 (0.90–2.61)	0.12
ICU admission		
No	Referent	
Yes	1.70 (1.23–2.36)	0.001
Receipt of systemic antifungal treatment		
No	Referent	
Yes	0.35 (0.25–0.48)	<0.001
*Candida* species		
* C. albicans*	Referent	
Other *Candida* spp.	0.66 (0.49–0.89)	0.006

*aOR, adjusted odds ratio; ICU, intensive care unit. Intra-cluster correlation coefficient = 0.03; likelihood ratio test for ρ = 0; p value = 0.003.

**Table 4:** Interaction between HIV and intensive care unit admission on in-hospital death among 907 persons with candidemia, adjusted for potential confounders, South Africa, 2012–2017*

Variable	Stratum-specific aOR for death (95% CI)	Wald test p value†
Admitted to ICU		
HIV-seronegative	Referent	
HIV-seropositive	1.56 (1.00–2.43)	0.05
Not admitted to ICU		
HIV-seronegative	Referent	
HIV-seropositive	2.27 (1.47–3.52)	<0.001

*aOR, adjusted odds ratio; ICU, intensive care unit.  †Overall likelihood ratio test for interaction p value = 0.22. Case-fatality ratio for those admitted to an ICU: HIV-seropositive (94/152; 62%), HIV-seronegative (157/301; 52%) versus those not admitted to an ICU: HIV-seropositive (117/224; 52%), HIV-seronegative (76/230; 33%).

### Kaplan-Meier Survival Analysis

**Figure 2:**
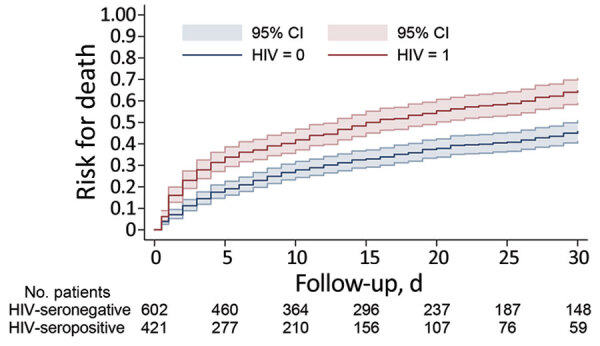
Kaplan-Meier analysis for 1,023 participants with candidemia during a 30-day period after the diagnosis of culture-confirmed candidemia by HIV infection status (outcome date missing for 17 participants), South Africa, 2012–2017. HIV = 0: HIV-seronegative; HIV = 1: HIV-seropositive; p value for log-rank test <0.001.

An outcome date was available for 1,023 participants. Overall, 44% (452/1,023) died within 30 days. The Kaplan-Meier survival curves diverged for HIV-seropositive and HIV-seronegative persons within 3 days of candidemia diagnosis and then remained roughly parallel until day 30 (Figure 2). Evidence was strong against the hypothesis that survival experience did not differ by HIV status (p<0.001).

### Association of HIV Status and ICU Admission

Among 1,023 participants, a lower proportion (175/422, 41%) of HIV-seropositive persons than HIV-seronegative persons (339/601, 56%) were admitted to the ICU (crude OR 0.55, 95% CI 0.42–0.71; p<0.001). After adjustment for sentinel site, age, sex, and quick Pitt score category (n = 583) (Appendix Table 5), HIV-seropositive participants were 60% less likely to be admitted to the ICU than HIV-seronegative participants (OR 0.40, 95% CI 0.25–0.64; p<0.001). Among HIV-seropositive participants, a similar proportion with and without advanced HIV disease were admitted to the ICU (72/166 [43%] vs. 39/99 [39%]; p = 0.53). A similar proportion of HIV-seropositive participants receiving and not receiving antiretroviral treatment were admitted to ICU (116/299 [39%] vs. 50/101 [50%]; p = 0.06).

## Discussion

In this surveillance study of hospitalized persons with candidemia in South Africa, the prevalence of HIV infection was 41% (95% CI 38%–44%). The 30-day mortality rate was almost twice as high among HIV-seropositive persons as among HIV-seronegative persons (OR 1.89, 95% CI 1.38–2.60; p<0.001), after adjusting for relevant confounders. The effect of HIV infection on death rates was estimated to be stronger among those not managed in an ICU than those who were, though the 95% CIs overlapped. HIV-seropositive persons also had a substantially lower risk for ICU treatment during their admission than those who were HIV-seronegative.

The overall crude mortality rate associated with candidemia of 44% (95% CI 41%–47%) reported in this study is higher than that reported in resource-rich settings (*19*), possibly owing to differences in our hospitalized cohort. The overall median age was lower, a large proportion were critically ill, and amphotericin B deoxycholate and azoles were the mainstay of treatment, whereas few patients received echinocandins. In our large study, we found a crude mortality rate among HIV-seropositive patients of 54%, which is comparable to the 60% mortality rate reported in 2 case series (*6*,*7*). Our main results are consistent with a United States cohort study that found that HIV infection was associated with a 2-fold increased adjusted hazard of 30-day mortality (*9*). We believe that this association between HIV and death among persons with candidemia is biologically plausible. First, the mechanism for an increased risk for death among HIV-seropositive persons may operate at the individual level. Despite a large proportion having received previous antiretroviral treatment, the median CD4 count among hospitalized HIV-seropositive participants with candidemia in this study was 133 cells/µL, and almost two thirds had advanced HIV disease (defined by the WHO as a CD4 count of <200 cells/µL) (*20*). This finding is consistent with reports of virologic failure and treatment interruption among an increasing proportion of HIV-seropositive patients at acute-care hospitals in South Africa (*21*). Many persons with advanced HIV disease have neutropenia or neutrophil defects (*10*,*11*). This innate immune defect may be a consequence of abnormal progenitor stem cell growth, a deficiency of granulocyte colony-stimulating factor, bone marrow infiltration by opportunistic infectious agents or malignant cells, treatment with particular medicines, or autoimmune phenomena (*10*). The alteration in the innate immune response may then reduce the clearance of *Candida* from the bloodstream, despite appropriate antifungal treatment. People with advanced HIV disease might also have been admitted primarily for management of disseminated tuberculosis, cryptococcal disease, or *Pneumocystis* pneumonia, which are associated with high death rates (*20*). In addition, more than one third of HIV-seropositive participants in this study were recorded to have evidence of HIV-associated wasting. Patients with a poorer nutritional state or loss of lean body mass have worse outcomes (*22*).

Second, an increased risk for death may be a consequence of differential treatment practices at the sentinel hospitals for HIV-seronegative and HIV-seropositive persons. We found that HIV-seropositive persons were 60% less likely to have received intensive care during their hospitalization. Several studies in Italy have documented the large proportion of patients who are managed for candidemia in general medical wards (*23*–*25*). In addition, the adjusted effect of HIV infection on 30-day mortality rates was stronger among participants who were not managed in an ICU during their hospital stay. This finding suggests that intensive care and monitoring of patients with candidemia, including those who are HIV-seropositive, might reduce mortality rates. However, in a resource-limited setting, the number of ICU beds is restricted. The criteria for admission to a public-sector ICU in South Africa includes an assessment of the severity of the acute or underlying illness and whether organ dysfunction can realistically be reversed. These criteria apply equally to those with and without HIV infection, according to a survey of critical care physicians in South Africa, although no data exist on the proportion of HIV-seropositive persons who were eligible for ICU admission but were not referred or were turned down (*26*,*27*). Among HIV-seropositive participants in our study, similar proportions of persons with advanced HIV disease who were receiving antiretroviral treatment were or were not admitted to ICU, suggesting that these factors were not the sole criteria for admission.

We confirmed several well-described risk factors for death among patients with candidemia, including inappropriate or no antifungal treatment, ICU admission, increased age, and a quick Pitt score of >2 (*28*). Removal of a CVC following diagnosis of candidemia was protective against death. A limitation of this study is that it was a secondary data analysis and was not specifically designed or powered to answer whether HIV infection was associated with death among patients with candidemia. However, the study was conducted in a high HIV prevalence setting, our sample size was large, and we found strong and consistent evidence against the null hypothesis after adjustment for confounding. Selection bias was a limitation because we excluded 60% of cases diagnosed at sentinel hospitals for whom data for the main exposure and outcome variables were missing. We also found differences among those who were included and excluded from the analysis. For instance, we included a larger proportion of cases among adults 18–44 years of age, cases caused by *C. albicans*, and cases from outside Gauteng Province in the analysis (Appendix). Data were also missing for confounder variables, and we excluded several potentially critical confounders from the main multivariable analysis. However, including these variables in a smaller dataset for multivariable analysis did not change the point estimate for the main exposure effect, although the 95% CI was wider (Appendix Table 3). In ≈20% of cases, HIV status was self-reported by participant or next-of-kin interview if a clear record of HIV status was not in the chart. If HIV-seropositive persons underreported their actual infection status, it might have weakened the association with death demonstrated in this study. Because we were unable to adjust for unmeasured factors, such as the presence or severity of diagnosed or undiagnosed underlying conditions, residual confounding is also possible. We conducted this study in an upper middle-income country, and participants were recruited at urban sentinel hospitals with ICU facilities; therefore, the results might not apply to all hospital populations. Cause of death was not recorded, and we were thus unable to estimate the number of deaths directly attributable to candidemia among either HIV-seropositive or HIV-seronegative participants. We excluded children <18 months of age from this analysis and cannot comment on whether HIV exposure or infection is associated with death in this population.

A key strength of this study was that it was nested within a large active surveillance system; laboratory audits were conducted to ensure that all culture-confirmed cases were captured. The main outcome measure was in-hospital death, a clear endpoint that was unlikely to have been misclassified. Given that most persons with HIV infection live in sub-Saharan Africa and the risk for healthcare-associated infections, including those caused by antimicrobial-resistant fungi, is becoming increasingly critical in this region (*1*,*29*–*31*), our findings might have broader implications than similar studies and are an essential addition to the literature.

In conclusion, we found that the overall crude mortality rate associated with candidemia was high, and HIV-seropositive persons were at ≈2-fold increased adjusted risk for all-cause in-hospital death, compared with their HIV-seronegative counterparts. This effect on mortality rates was weakened among those admitted to the ICU, though HIV-seropositive persons were substantially less likely to have received intensive care. We recommend a high index of suspicion for candidemia among admitted HIV-seropositive persons, regardless of the presence of classical risk factors. We further recommend that HIV-seropositive persons with suspected candidemia rapidly begin appropriate early antifungal treatment, that they be investigated to identify an infection source and control measures instituted, and that they should be considered for intensive care and monitoring to reduce deaths. Where feasible, consultation with an infectious disease specialist would enable this level of care.

Appendix Additional information about HIV infection as risk factor for death among hospitalized persons with candidemia, South Africa, 2012–2017.
