# Supplementary material for: HIV Infection as Risk Factor for Death among Hospitalized Persons with Candidemia, South Africa, 2012–2017
Source: Emerg Infect Dis. 2021 Jun;27(6):1607–15. doi: 10.3201/eid2706.210128 (PMC8153852; doi:10.3201/eid2706.210128)
Supplement: Appendix — Additional information about HIV infection as risk factor for death among hospitalized persons with candidemia, South Africa, 2012–2017. [file 21-0128-Techapp-s1.pdf]

Article DOI: https://doi.org/10.3201/eid2706.210128

HIV Infection as Risk Factor for Death among Hospitalized Persons with Candidemia, South Africa, 2012–2017

Appendix

Supplementary Methods

Sentinel Hospital and Diagnostic Laboratory Practices

Attending clinicians initiated empiric antifungal treatment when an episode of candidemia was suspected. Amphotericin B deoxycholate and fluconazole was widely available, but echinocandin treatment was limited to private-sector hospitals and academic public-sector hospitals. Blood culture specimen collection practices varied widely among provinces and between the private and public sector (*1*). Diagnostic laboratories serving the sentinel hospitals used automated blood culturing systems such as Bactec (BD, https://www.bd.com) and processed specimens by using standard operating procedures.

Confounding and Interaction

We regrouped potential confounder variables into clinically meaningful categories. Intensive care unit (ICU) admission was recorded as a binary variable as a response to the question: Has the participant ever been admitted for intensive care during this hospital admission? The Pitt bacteremia score, a marker of disease severity, is as accurate as the APACHE II score for predicting death among critically ill persons with sepsis and has also been shown to be associated with death among persons with candidemia (*2*,*3*). We calculated a quick Pitt score, an abbreviated version of the Pitt bacteremia score, as the sum of individual scores for body temperature of <35°C (1 point), systolic blood pressure of <90 mm Hg (1 point), cardiac arrest (1 point), mechanical ventilation (1 point), and altered mental status (1 point) on the day of diagnosis of candidemia. A quick Pitt score of ≥2 was strongly correlated with a Pitt bacteremia score of ≥4 and predicted death in patients with carbapenem-resistant Enterobacterales infections (*4*). Antifungal resistance was defined on the basis of Clinical and Laboratory Standards Institute

M60 *Candida* species–specific breakpoints for MICs (MIC); this was restricted to species or antifungal agents for which breakpoints have been published (*5*). Inappropriate antifungal treatment was defined as treatment around the time of diagnosis with an antifungal agent to which the *Candida* species was resistant. We defined a mixed episode of candidemia as >1 *Candida* spp. either cultured from a single blood culture specimen or from multiple specimens over the 30-day period. We defined community-onset infection as a positive blood culture within 72 hours of hospital admission. We considered age and sex to be important a priori confounders. We screened potential confounders by cross-tabulating these variables with the main exposure and looked for associations in our dataset. None of the potential confounders were considered to be on the causal pathway for the main study question. We decided that the following potential confounding variables had collinear relationships with the main exposure: ICU admission, central venous catheter in situ, and total parenteral nutrition. We chose to use ICU admission in our models.

Classical Analyses

We looked for evidence that the effect estimate for the association between HIV status and 30-day mortality was confounded or modified by each explanatory variable. We compared the summary weighted odds ratio (OR) to the crude OR and looked for a ≈10% change in main exposure effect estimate as evidence of confounding. We also visually compared the stratum-specific ORs across each level of the potential effect-modifying variable and performed a test of homogeneity of ORs.

Model Building

We omitted potential confounders with a large amount of missing data. We checked the number of events and then applied a rule of ten (events per variable) to ensure that the models included a reasonable number of parameters. We forced the prespecified confounder variables (i.e., age, sex) into a baseline model and then added other potential confounders, one at a time using a forward approach, starting with the variable with the strongest association with death on univariable analysis. We retained a potential confounder in the model if this changed the main effect estimate by 10% and then added the other variables in turn. We checked the reliability of parameter estimates by using the quadchk command in Stata (https://www.stata.com) because the number of participants within many of the 29 clusters was large (>20). We compared the

final logistic regression models with and without an interaction term between HIV status and ICU admission.

Supplementary Results

Description of 1,040 Cases Included in Analysis

The number of sentinel sites and thus the number of cases varied by year of surveillance: 2012 (9 sites in Gauteng/Western Cape, 139 cases), 2013 (10 sites in Gauteng/Western Cape, 185 cases), 2014 (17 sites in other 7 provinces, 100 cases), 2015 (20 sites in other 7 provinces, 91 cases), 2016 (25 sites in all provinces, 273 cases), 2017 (27 sites in all provinces, 252 cases). All sites were public-sector hospitals except 3 private hospital sites that participated in 2016 and 2017. We conducted a complete records analysis. Among 1,040 cases included in this analysis, 730 (70%) case report forms were completed by interview plus medical chart review versus 310 (30%) by retrospective medical chart review alone. In general, the completeness of case report forms was better for participants who were interviewed and had their medical charts prospectively reviewed. Among the 806 cases that were excluded because of missing HIV status or outcome data, 374 (46%) had case report forms completed by interview or medical chart review versus 432 (54%) by medical chart review alone ($p<0.001$). The median time of survival among 307 participants who did not receive systemic antifungal treatment was 3 days (interquartile range [IQR] 1–10 days) from the positive blood culture date versus 16 days (IQR 8–32 days) among 686 who received treatment.

Comparison of HIV-Seropositive and HIV-Seronegative Persons

Compared with 614 HIV-seronegative participants with candidemia, a larger proportion of their HIV-seropositive counterparts were 18–44 years of age (237/425 [56%] vs. 190/612 [31%]; $p<0.001$), female (226/426 [53%] vs. 272/614 [44%]; $p = 0.005$), received a diagnosis later in the surveillance period (2015–2017) (263/426 [62%] vs. 353/614 [57%]; $p<0.001$), not admitted to an ICU (247/422 [59%] vs. 262/601 [44%]; $p < 0.001$), not receiving total parenteral nutrition (334/412 [81%] vs. 425/592 [72%]; $p<0.001$), not receiving fluids or medicines through a central venous catheter (CVC) (223/414 [54%] vs. 243/597 [41%]; $p < 0.001$), infected with *C. albicans* (204/389 [52%] vs. 221/557 [40%]; $p<0.001$), not infected with an antifungal-resistant *Candida* spp. (265/305 [87%] vs. 345/459 [75%]; $p<0.001$) and not receiving systemic

antifungal treatment around the time of candidemia diagnosis (157/415 [38%] vs. 153/595 [26%]; p<0.001) (Table 1 in the main text). A similar proportion of HIV-seropositive and HIV-seronegative persons had recorded evidence of complications of candidemia (deep organ involvement) in their medical charts (17/426 [4%] vs. 28/614 [5%]; p = 0.66).

Unadjusted Risk Factors for 30-Day Mortality

The case fatality ratio was incrementally higher in each of the 3 older age groups compared with the baseline category (i.e., 18 months–17 years) (p<0.001) (Table 2 in the main text). ICU admission was associated with a 43% increased crude risk for death (95% CI 1.11–1.83; p = 0.005). For 652 participants with a quick Pitt score, a score of ≥2 was associated with a 2.49 increased risk for 30-day mortality (95% CI 1.71–3.61; p<0.001). Compared with *C. albicans* infection, infection with a species other than *C. albicans* was associated with a 41% reduced crude odds of death (95% CI 0.46–0.77; p<0.001). Receipt of systemic antifungal treatment around the time of candidemia diagnosis was associated with a 63% reduced odds of 30-day mortality (95% CI 0.28–0.49; p<0.001). Among 488 participants with available data, removal of an indwelling CVC was protective against death (OR 0.51, 95% CI 0.34–0.76; p<0.001).

Classical Stratified Analysis of the Relationship between HIV Status and 30-Day Mortality

Upon classical stratified analysis, the summary odds of death at 30 days remained approximately 2-fold higher among HIV-seropositive participants versus HIV-seronegative participants with candidemia when adjusted for each potential confounder in turn. We found consistently strong evidence against the null hypothesis of an OR = 1 (p<0.001 in all cases, except when adjusting for inappropriate antifungal treatment) (Appendix Table 2). ICU admission was a negative confounder of the association between HIV and 30-day mortality; the point estimate for the summary mortality OR shifted by >10% from 1.89 (95% CI 1.38–2.60) to 2.09 (95% CI 1.60–2.71) after adjusting for this variable. A quick Pitt score of ≥2 was also a negative confounder; the point estimate changed to 2.28 (95% CI 1.63–3.18) after adjustment. Conversely, the summary OR point estimate shifted ≈10% lower to 1.72 (95% CI 1.13–2.61) when we adjusted for inappropriate antifungal treatment. However, as already noted, we had a large amount of missing data for both the latter variables.

Using a low-power test for interaction, we did not find strong evidence of an interaction between HIV status and any other variable when comparing 30-day mortality rates among HIV-seropositive persons with those of HIV-seronegative persons. Conversely, upon visual inspection of the stratum-specific rate ratios in Appendix Table 2, we identified the following as possible effect modifiers (different stratum-specific OR point estimates but overlapping 95% CI): age, sex, year of diagnosis, ICU admission, inappropriate antifungal treatment, and removal of the CVC. However, we focused only on ICU admission as an effect modifier because we had specified this a priori.

Random-Effects Multivariable Logistic Regression Analysis

We conducted a 2-level random-effects multivariable logistic regression analysis. We excluded 133 cases with missing data for any of the confounders; thus, the final model included 907 participants. We excluded quick Pitt score and inappropriate antifungal treatment from the final analysis because there were large amounts of missing data, even though these were potentially important confounders. However, when these variables were retained in a model with fewer observations (n = 263), the point estimate for the effect of HIV infection on mortality rates was very similar to that described in the final model (Appendix Table 3).

Dose-Response Effect of HIV Infection

We considered HIV status as an ordinal variable: HIV-seronegative, HIV-seropositive without advanced immunosuppression (CD4 count ≥200 cells/µL), and HIV-seropositive with advanced immunosuppression (CD4 count <200 cells/µL). We then adjusted for sentinel hospital, age, sex, year of diagnosis, ICU admission, receipt of systemic antifungal treatment, and *Candida* species in a 2-level random effects model (n = 767) (Appendix Table 4). Compared with mortality among HIV-seronegative persons, the adjusted odds of 30-day mortality was 1.90 times higher among HIV-seropositive persons without advanced immunosuppression (95% CI 1.13–3.20; p = 0.02) and 2.18 times higher among persons with advanced HIV disease (95% CI 1.39–3.42; p = 0.001). After adjustment for sentinel site, age, sex, and quick Pitt score category (n = 583) (Appendix Table 5), HIV-seropositive persons had a 60% reduced risk for ICU admission compared with HIV-seronegative participants (OR 0.40, 95% CI 0.25–0.64; p < 0.001).

Supplementary Discussion

In 2 small studies of HIV-seropositive participants with candidemia, infection with a species other than *C. albicans* was independently associated with death (*6*,*7*). On the basis of these previous reports, we initially hypothesized that HIV-seropositive persons would be more likely to be infected with a species other than *C. albicans*. Since these species are more likely to be antifungal-resistant, we then speculated that death would be more likely in this group because of inappropriate antifungal treatment. However, we found that HIV-seropositive participants had a 34% lower odds of being infected with *Candida* species other than *C. albicans.* This is also consistent with our finding that almost a third of HIV-seropositive persons may have acquired candidemia as a community-onset infection, though a similarly large proportion of HIV-seronegative persons also had a diagnosis within 72 hours of admission. We did not collect information on all healthcare contacts in the preceding 12 months so could not exclude that these infections were healthcare-associated. Community-onset infections in HIV-seropositive persons have been documented in a single-center study in South Africa (*8*). Despite its susceptibility to most antifungal agents, *C. albicans*, a common component of the gastrointestinal microbiota, has a well-described array of virulence factors that may increase severity of illness and risk for death (*9*). In a study in Italy, persons with candidemia caused by >1 *Candida* spp. versus monomicrobial infection were more likely to be HIV-seropositive (2/15 vs. 14/737). In turn, mixed candidemia was independently associated with increased deaths; the authors hypothesized that this effect was mediated by a larger fungal bloodstream burden in mixed infections (*10*). In our study, only 3% of patients had a mixed infection, and proportions were similar in HIV-seropositive groups and HIV-seronegative groups.

References

1. Abrahams MS, Whitelaw AC, Orth H. Time for a culture change? Suboptimal compliance with blood culture standards at a district hospital in Cape Town. S Afr Med J. 2015;105:1039–43. PubMed https://doi.org/10.7196/SAMJ.2015.v105i12.9442

2. Rhee JY, Kwon KT, Ki HK, Shin SY, Jung DS, Chung DR, et al. Scoring systems for prediction of mortality in patients with intensive care unit-acquired sepsis: a comparison of the Pitt bacteremia score and the Acute Physiology and Chronic Health Evaluation II scoring systems. Shock. 2009;31:146–50. PubMed https://doi.org/10.1097/SHK.0b013e318182f98f

3. Vaquero-Herrero MP, Ragozzino S, Castaño-Romero F, Siller-Ruiz M, Sánchez González R, García-Sánchez JE, et al. The Pitt Bacteremia Score, Charlson Comorbidity Index and Chronic Disease Score are useful tools for the prediction of mortality in patients with *Candida* bloodstream infection. Mycoses. 2017;60:676–85. PubMed https://doi.org/10.1111/myc.12644

4. Henderson H, Luterbach CL, Cober E, Richter SS, Salata RA, Kalayjian RC, et al. The Pitt Bacteremia Score predicts mortality in non-bacteremic infections. Clin Infect Dis. 2020;70:1826–33. **PMID 31219148**

5. Clinical and Laboratory Standards Institute. Performance standards for antifungal susceptibility testing of yeasts (M60). Wayne (PA): The Institute; 2017.

6. Tumbarello M, Tacconelli E, de Gaetano Donati K, Morace G, Fadda G, Cauda R. Candidemia in HIV-infected subjects. Eur J Clin Microbiol Infect Dis. 1999;18:478–83. PubMed https://doi.org/10.1007/s100960050327

7. Bertagnolio S, de Gaetano Donati K, Tacconelli E, Scoppettuolo G, Posteraro B, Fadda G, et al. Hospital-acquired candidemia in HIV-infected patients. Incidence, risk factors and predictors of outcome. J Chemother. 2004;16:172–8. PubMed https://doi.org/10.1179/joc.2004.16.2.172

8. Kreusch A, Karstaedt AS. Candidemia among adults in Soweto, South Africa, 1990-2007. Int J Infect Dis. 2013;17:e621–3. PubMed https://doi.org/10.1016/j.ijid.2013.02.010

9. Noble SM, Gianetti BA, Witchley JN. *Candida albicans* cell-type switching and functional plasticity in the mammalian host. Nat Rev Microbiol. 2017;15:96–108. PubMed https://doi.org/10.1038/nrmicro.2016.157

10. Ramos A, Romero Y, Sánchez-Romero I, Fortún J, Paño JR, Pemán J, et al. Risk factors, clinical presentation and prognosis of mixed candidaemia: a population-based surveillance in Spain. Mycoses. 2016;59:636–43. PubMed https://doi.org/10.1111/myc.12516

Appendix Table 1. Characteristics of 2,563 patients with candidemia at sentinel sites who were included or excluded from analysis of HIV infection as risk factor for death, South Africa, 2012–2017

		Excluded from analysis		Included in analysis	
Variable	No. patients	Case report form not completed (n = 717)	Case report form completed but missing HIV status or outcome data (n = 806)	Case report form completed including HIV status and outcome data (n = 1040)	p value*
Age group, y					
<18	478	58 (12)	194 (41)	226 (47)	<0.001
18–44	742	126 (17)	189 (25)	427 (58)	
45–64	652	145 (22)	224 (34)	283 (43)	
≥65	389	113 (29)	175 (45)	101 (26)	
Missing	302	275 (91)	24 (8)	3 (1)	
Sex					
F	1,003	167 (16)	338 (34)	498 (50)	0.04
M	1,199	191 (16)	466 (39)	542 (45)	
Missing	361	359 (99)	2 (1)	0 (0)	
Year					
2012	330	142 (43)	49 (15)	139 (42)	<0.001
2013	321	61 (19)	75 (23)	185 (58)	
2014	161	17 (11)	44 (27)	100 (62)	
2015	162	14 (9)	57 (35)	91 (56)	
2016	754	249 (33)	232 (31)	273 (36)	
2017	835	234 (28)	349 (42)	252 (30)	
Missing	0	0 (0)	0 (0)	0 (0)	
Province					
Other province	845	97 (11)	253 (30)	495 (59)	<0.001
Gauteng	1,715	617 (36)	553 (32)	545 (31)	
Missing	3	3 (100)	0 (0)	0 (0)	
Candida species					
Candida albicans	790	109 (14)	256 (32)	425 (54)	<0.001
Other *Candida* species	1,504	517 (34)	466 (31)	521 (35)	
Missing	269	91 (34)	84 (31)	94 (35)	

*P value by Pearson's χ^2 test (excluding missing category) to compare 3 groups.

Appendix Table 2. Effect of HIV infection on 30-day mortality rate among 1,040 persons with candidemia, adjusted in turn for each potential confounder, South Africa, 2012–2017*

Variable	No. patients	Stratum-specific OR (95% CI)	Mantel-Haenszel summary OR (95% CI)	Score test p value	p value for test of interaction
Age group, y					
<18	226	1.11 (0.54–2.25)	2.03 (1.54–2.68)	<0.001	0.21
18–44	427	2.61 (1.73–3.92)			
45–64	283	1.94 (1.18–3.19)			
≥65	101	1.56 (0.44–5.50)			
Sex					
F	498	1.56 (1.09–2.23)	1.94 (1.50–2.50)	<0.001	0.09
M	542	2.42 (1.68–3.48)			
Year					
2012	139	1.31 (0.63–2.75)	1.85 (1.43–2.39)	<0.001	0.21
2013	185	1.59 (0.86–2.93)			
2014	100	2.44 (1.06–5.59)			
2015	91	0.80 (0.35–1.86)			
2016	273	2.29 (1.37–3.84)			
2017	252	2.41 (1.43–4.05)			
ICU admission					
No	509	2.46 (1.69–3.58)	2.09 (1.60–2.71)	<0.001	0.21
Yes	514	1.76 (1.21–2.56)			
CVC in situ					
No	466	1.95 (1.33–2.84)	2.05 (1.57–2.67)	<0.001	0.71
Yes	545	2.15 (1.49–3.10)			
Total parenteral nutrition					
No	759	1.90 (1.42–2.55)	1.98 (1.53–2.58)	<0.001	0.55
Yes	245	2.31 (1.32–4.04)			
Quick Pitt score of ≥2†					
No	503	2.28 (1.57–3.33)	2.28 (1.63–3.18)	<0.001	0.98
Yes	149	2.26 (1.11–4.62)			
Candida species					
Candida albicans	425	1.98 (1.34–2.93)	1.74 (1.33–2.28)	<0.001	0.37
Other *Candida* species	521	1.55 (1.07–2.24)			
Candida species resistant to fluconazole, voriconazole or an echinocandin‡					
No	610	1.97 (1.42–2.73)	1.93 (1.43–2.61)	<0.001	0.78
Yes	154	1.76 (0.84–3.66)			
Receipt of systemic antifungal treatment					
No	310	1.95 (1.22–3.12)	1.78 (1.37–2.32)	<0.001	0.64
Yes	700	1.71 (1.24–2.35)			
Inappropriate antifungal treatment for candidemia§					
No	381	1.78 (1.15–2.75)	1.72 (1.13–2.61)	0.01	0.58
Yes	38	1.13 (0.25–5.23)			
Removal of CVC					
No	127	2.57 (1.20–5.53)	1.99 (1.35–2.94)	<0.001	0.44
Yes	361	1.81 (1.15–2.85)			

*CVC, central venous catheter; ICU, intensive care unit; OR, odds ratio.
†Quick Pitt score was calculated as the sum of individual scores for temperature <35°C (1), systolic blood pressure <90 mm Hg (1), cardiac arrest (1), mechanical ventilation (1), and altered mental status (1) at time of diagnosis of candidemia.
‡Resistance was defined on the basis of Clinical and Laboratory Standards Institute M60 *Candida* species-specific breakpoints.
§Inappropriate treatment was defined as treatment with an antifungal agent to which the *Candida* species was resistant.

Appendix Table 3. Random-effects multivariable logistic regression analysis of the effect of HIV on 30-day mortality rates by potential confounder among 263 persons with candidemia, South Africa, 2012–2017*

Variable	Summary aOR for death (95% CI)	Wald p value
HIV status		
Seronegative	1	
Seropositive	1.79 (0.96–3.35)	<0.07
Age group, y		
<18	1	
18–44	1.62 (0.70–3.75)	0.26
45–64	3.11 (1.30–7.45)	0.01
≥65	5.50 (1.96–15.48)	0.001
Sex		
F	1	
M	1.40 (0.80–2.43)	0.24
Year		
2012	1	
2013	1.35 (0.55–3.33)	0.51
2014	1.64 (0.57–4.74)	0.36
2015	0.82 (0.23–2.90)	0.76
2016	0.60 (0.23–1.60)	0.31
2017	1.83 (0.77–4.37)	0.17
ICU admission		
No	1	
Yes	1.10 (0.55–2.17)	0.80
Receipt of systemic antifungal treatment		
No	1	
Yes	Omitted from model	
Candida species		
Candida albicans	1	
Other *Candida* species	0.96 (0.53–1.73)	0.89
Quick Pitt score of ≥2†		
No	1	
Yes	1.64 (0.81–3.32)	0.17
Inappropriate antifungal treatment for candidemia‡		
No	1	
Yes	0.87 (0.31–2.40)	0.78

*Data sparsity in this model may affect estimates. Intracluster correlation coefficient<0.001; likelihood ratio test for ρ = 0; p value = 0.50. aOR, adjusted odds ratio; ICU, intensive care unit.
†Quick Pitt score was calculated as the sum of individual scores for temperature <35°C (1), systolic blood pressure <90 mm Hg (1), cardiac arrest (1), mechanical ventilation (1), and altered mental status (1) at time of diagnosis of candidemia.
‡Resistance was defined on the basis of Clinical and Laboratory Standards Institute M60 *Candida* species-specific breakpoints. Inappropriate treatment was defined as treatment with an antifungal agent to which the *Candida* species was resistant.

Appendix Table 4. Random-effects multivariable logistic regression analysis of the effect of HIV as an ordinal variable on 30-day mortality rates by potential confounder among 767 persons with candidemia, South Africa, 2012–2017*

Variable	Summary aOR for death (95% CI)	Wald p value
HIV status		
Seronegative	1	
Seropositive with CD4 count ≥200 cells/μL	1.90 (1.13–3.20)	0.02
Seropositive with CD4 count <200 cells/μL	2.18 (1.39–3.42)	0.001
Age group, y		
<18	1	
18–44	2.25 (1.38–3.67)	0.01
45–64	3.20 (1.93–5.31)	<0.001
≥65	5.65 (3.01–10.57)	<0.001
Sex		
F	1	
M	1.31 (0.95–1.82)	0.10
Year		
2012	1	
2013	0.97 (0.53–1.78)	0.92
2014	1.14 (0.53–2.43)	0.74
2015	1.05 (0.49–2.24)	0.89
2016	0.90 (0.50–1.62)	0.73
2017	1.48 (0.83–2.64)	0.19
ICU admission		
No	1	
Yes	1.92 (1.34–2.74)	<0.001
Receipt of systemic antifungal treatment		
No	1	
Yes	0.31 (0.21–0.46)	<0.001
Candida species		
Candida albicans	1	
Other *Candida* species	0.65 (0.47–0.90)	0.01

*Intracluster correlation coefficient = 0.04; likelihood ratio test for ρ = 0; p value = 0.003. aOR, adjusted odds ratio; ICU, intensive care unit.

Appendix Table 5. Random-effects multivariable logistic regression analysis of the effect of HIV infection on intensive care unit admission by sentinel site, age, sex, and quick Pitt score category among 583 persons with candidemia, South Africa, 2012–2017*

Variable	Summary aOR for ICU admission (95% CI)	Wald p value
HIV status		
Seronegative	1	
Seropositive	0.40 (0.25–0.64)	<0.001
Age group, y		
<18	1	
18–44	3.22 (1.72–6.04)	<0.001
45–64	2.51 (1.28–4.90)	0.007
≥65	3.44 (1.37–8.67)	0.009
Sex		
F	1	
M	0.84 (0.55–1.31)	0.46
Quick Pitt score of ≥2†		
No	1	
Yes	23.81 (11.57–49.02)	<0.001

*aOR, adjusted odds ratio; ICU, intensive care unit. Intracluster correlation coefficient = 0.39; likelihood ratio test for ρ = 0; p value<0.001.
†Quick Pitt score was calculated as the sum of individual scores for temperature <35°C (1), systolic blood pressure <90 mm Hg (1), cardiac arrest (1), mechanical ventilation (1), and altered mental status (1) at time of diagnosis of candidemia.
